# Effect of Different Wheat Sprouting Conditions on the Characteristics of Whole-Wheat Flour

**DOI:** 10.17113/ftb.62.02.24.8435

**Published:** 2024-06

**Authors:** José Luis Navarro, Pedro Losano Richard, Malena Moiraghi, Mariela Bustos, Alberto Edel León, María Eugenia Steffolani

**Affiliations:** 1 Institute of Food Science and Technology of Córdoba (ICYTAC), CONICET-UNC Valparaiso y Rogelio Martínez, 5000 Córdoba, Argentina; 2 Department of Biological Chemistry, Faculty of Agricultural Sciences, National University of Córdoba (UNC), Valparaiso y Rogelio Martínez, 5000 Cordoba, Argentina

**Keywords:** bioactive compounds, enzymatic activity, pasting properties, sprouted whole-wheat flour, techno-functional ingredients

## Abstract

**Research background:**

Controlled sprouting promotes physiological and biochemical changes in whole grains, improves their nutritional value and offers technological advantages for breadmaking as an alternative to traditional whole grains. The aim of this study is to find sprouting conditions for the grains of Klein Valor wheat variety (*Triticum aestivum* L.) that would increase the nutritional value without significantly affecting the gluten proteins, which are essential in wholegrain baked goods.

**Experimental approach:**

The chemical and nutritional composition, enzymatic activity and pasting properties of the suspensions of unsprouted and sprouted whole-wheat flour were evaluated.

**Results and conclusions:**

This bioprocess allowed us to obtain sprouted whole-wheat flour with different degrees of modification in its chemical composition. Sprouting at 25 °C resulted in an observable increase in enzymatic activity and metabolic processes, particularly α-amylases, which significantly affect the starch matrix and the associated pasting properties. Additionally, there was a smaller but still notable effect on the structure of the cell walls and the protein matrix due to the activation of endoxylanases and proteases. In contrast, sprouting at 15 and 20 °C for 24 h allowed for better process control as it resulted in nutritional improvements such as a higher content of free amino acid groups, free phenolic compounds and antioxidant capacity, as well as a lower content of phytates. In addition, it provided techno-functional advantages due to the moderate activation of α-amylase and xylanase. A moderate decrease in peak viscosity of sprouted whole-wheat flour suspensions was observed compared to the control flour, while protein degradation was not significantly prolonged.

**Novelty and scientific contribution:**

Sprouted whole-wheat flour obtained under milder sprouting conditions with moderate enzymatic activity could be a promising and interesting ingredient for wholegrain baked goods with improved nutritional values and techno-functional properties. This approach could avoid the use of conventional flour improvers and thus have a positive impact on consumer acceptance and enable the labelling of the product with a clean label.

## INTRODUCTION

Wheat is irreplaceable crop for breadmaking for the majority of the world’s population. Whole-wheat products have attracted great interest due to their nutritional benefits, such as dietary fibre, vitamins, antioxidants and other health-promoting compounds (*1*). Despite its benefits, the consumption of whole wheat remains below recommended amounts in many countries. Additionally, the incorporation of high fibre content in cereal-based products remains a technological challenge due to the need to maintain acceptable dough rheological properties and sensory attributes. Several pretreatments have been proposed to counteract the negative effects, such as particle size reduction (*2*), pearling (*3*), enzymatic treatment (*4*) and fermentation (*5*). In this context, the use of sprouting as a natural and cost-effective method to improve the nutritional value (*6*, *7*) and acceptability of whole grains (*1*) is of growing interest. This physiological process begins when the grains meet suitable environmental conditions: certain hydrolytic enzymes are activated and break down the storage macromolecules for the developing seedling (*8*). The amylases rapidly degrade the starch, making it more digestible and consequently increasing the bioavailability of the reducing sugars. However, excessive accumulation of hydrolytic enzymes, often seen in preharvest sprouting, is a negative aspect from a technological point of view, since it affects flour functionality. Xylanases degrade the arabinoxylans of cell walls, increasing the availability of micronutrients (*9*), and proteases hydrolyse proteins, increasing the availability of peptides and amino acids (*1*). The metabolic activity also promotes the biosynthesis of polyphenols and increases their antioxidant capacity (*8*). Additionally, this bioprocess increases phytase activity and improves the absorption of essential minerals in the gastrointestinal tract (*10*).

Recent studies have reported that controlled sprouting improved the breadmaking properties of common wheat in terms of specific volume, crumb structure and softness during storage when added to white bread (*11*–*13*). These positive effects were attributed to the natural enzymes, which could decrease or completely replace the quantity of commercial enzymes frequently added to bread formulation (*11*, *14*). In addition, better sensory attributes like flavour and taste were also reported after the use of sprouted grains (*15*). In this way, careful control of sprouting conditions is necessary to achieve a good balance between the nutritional benefits and technological performance of wheat flour for breadmaking (*9*). Sprouted flour could also be considered as whole grain flour according to the definition of the Whole Grains Council (*16*), if all bran, germ, endosperm and vegetative parts are preserved as long as sprout growth does not exceed the grain length.

It is well-known that whole-wheat flour has functional deficiencies compared to refined flour due to the dilution of gluten by a high content of insoluble arabinoxylans, which disrupt the gluten network, and the presence of phytates, which have a negative effect on the bioavailability of minerals. Therefore, understanding the effects of controlled sprouting on protein aggregation, soluble fibre and phytic acid content in wheat is of utmost importance. For this reason, this study investigates the effect of controlled wheat sprouting on the chemical composition, hydrolytic enzyme activities and pasting properties of the resulting sprouted whole-wheat flour. The aim is to investigate the effects of sprouting on various nutritional and technological properties, which provide essential elements for the production of wholegrain bread with improved nutritional value and good technological quality.

## MATERIALS AND METHODS

### Materials and chemicals

We used the commercial wheat variety Klein Valor (*Triticum aestivum* L.) and grains from the same lot sprouted in the laboratory under controlled conditions. The wheat was provided by the Estación Experimental Agropecuaria Marcos Juárez del INTA (Córdoba, Argentina), harvested in 2020 and originated from a cultivation area with the following geographical coordinates: 32° 43’ S, 62° 6’ W. Klein Valor variety is a durum wheat with very strong gluten network according to the genetic quality classification established by the Winter Cereal Committee of the National Seed Commission (Argentina) (*17*). All chemicals used in this study were of analytical grade.

### Sprouting conditions

Wheat grains were first surface sterilised in a 1 % sodium hypochlorite solution for 5 min. The grains were then steeped in potable tap water at 18 °C for 24 h (1:2 water ratio). The drained samples were placed in trays and put in a germination chamber (Servicios Mecatrónicos, Córdoba, Argentina) at different temperatures (15, 20 and 25 °C) and a relative humidity of 95 % in the dark. Samples for analysis were taken after 18, 24 and 48 h. The degree of sprouting (DoS) of 100 grains and their average value were determined visually by classifying the length of the coleoptile and the radicles of the grains (0-7) according to Krapf *et al.* (*18*). The wheat sprouting conditions were selected based on preliminary tests (*19*). The sprouted grains were dried in an oven with air circulation at 50 °C for 20 h to limit the amount of water for enzyme activity. Finally, the sprouted grains (including the vegetable parts) were milled in a cyclonic mill (Cyclotec^TM^ 1093; Foss, Hillerød, Denmark) using a 1-mm mesh sieve to obtain sprouted whole-wheat flour. Unsprouted whole-wheat flour under the same milling and granulometry conditions was used as a control.

### Chemical characterisation of the flour samples

The moisture, protein (N×5.7), lipid and ash mass fractions of the unsprouted and sprouted whole-wheat flour samples were determined according to AACC standard methods 44-15.02 (*20*), 46-12.01 (*21*), 30-26.01 (*22*) and 08-01.01 (*23*), respectively. Briefly, the moisture mass fraction was determined by weighing the sample before and after drying for 2 h at 130 °C (drying oven model 33 600 D060602; Memmert GmbH, Schwabach, Germany). The protein mass fraction (Kjeldahl method) was determined after digestion with concentrated H_2_SO_4_ (Sintorgan, Buenos Aires, Argentina). The digestor (model MB-6; RAYPA, Barcelona, Spain) was used for sample digestion and Kjeldahl distillation unit (VELP UDK126A; VELP Scientifica Srl, Milan, Italy) for distillation. Total lipids were determined by Soxhlet extraction with petroleum ether (Sintorgan). After the extraction, the lipid mass fraction was determined by weighing. The ash mass fraction was determined by weighing the sample before and after ignition at 600 °C for 2 h (model 332; Indef, Córdoba, Argentina). Total starch mass fraction was determined according to AACC method 76-13.01 (*24*) using a total starch assay kit (Megazyme Ltd, Wicklow, Ireland) at 510 nm with a UV-Vis spectrophotometer (model V-730; JASCO, Easton, MD, USA) taking into account the reducing sugar content of each sample to correct the results. The reducing sugar mass fraction was determined using 3,5-dinitrosalicylic acid (DNS) method described by Bustos *et al*. (*25*) at 545 nm with a UV-Vis spectrophotometer (model V-730; JASCO). Soluble sugars were extracted and determined using an HPLC-RI system (Shimadzu, Kyoto, Japan) equipped with a Luna® Omega 3 µm SUGAR 100 Å column (Phenomenex, Torrance, CA, USA) according to the modified method of Losano Richard *et al*. (*26*). The components were eluted with *φ*(acetonitrile)=75 % as the mobile phase at a flow rate of 1.2 mL/min. The oven temperature was 35 °C. The total run time was 25 min/sample. Quantification was based on a standard curve generated using commercial standards at five different concentrations. The content of water-soluble pentosans was quantified using the orcinol-HCl method described by Lancetti *et al*. (*27*) at 670 nm with UV-Vis spectrophotometer (model V-730; JASCO). All the analyses were carried out at least in duplicate and the concentrations were expressed on dry matter basis.

### Enzymatic activity of flour samples

#### α-amylase activity

The α-amylase activity of the flour samples was measured using the Amylazyme method (Megazyme, Bray, Ireland) (*28*). The unsprouted and sprouted whole-wheat flour samples (0.2 g) were accurately weighed into glass test tubes and incubated at 60 °C with sodium maleate buffer (5.0 mL, 100 mM, pH=6.0, 5 mM CaCl_2_). After 5 min of continuous stirring, an Amylazyme tablet was added. The reaction was allowed to continue for exactly 5 min and was stopped with Tris solution (6.0 mL, 2 % *m*/*V*, pH=9.0) under vigorous stirring on a vortex mixer. After filtration through a filter paper, the filtrate absorbance was measured at 590 nm (spectrophotometer model V-730; JASCO) against a reaction blank prepared by adding Tris solution before the Amylazyme tablet. Activities were calculated from the standard curve supplied with the Amylazyme kit (Megazyme) and expressed in α-amylase units (AU) per gram.

#### Endoxylanase activity

Endoxylanase activity in flour samples was measured using XylX6 method (Megazyme) according to Mangan *et al*. (*29*). To obtain the enzyme extracts, 50 mL of sodium acetate buffer solution (100 mM, pH=4.5) were added to the unsprouted and sprouted whole-wheat flour samples (1 g) in an Erlenmeyer flask and stirred for 15 min at room temperature. The resulting suspensions were clarified by centrifugation (1000×*g*, 10 min) and then diluted until a concentration of endoxylanase suitable for the assay was reached. Aliquots of the diluted extract solution were analysed for xylanase activity as follows: aliquots (0.05 mL) of the extract solutions were incubated with XylX6 reagent (0.05 mL) at 40 °C and the reactions were terminated after 10 min by the addition of Tris buffer (1.5 mL, 2 % *m*/*V*, pH=10.0). The absorbance of the reaction solutions and the reagent blank was measured at 400 nm (spectrophotometer model V-730; JASCO). The activities are expressed in xylanase units (XU) per gram.

### Effect of controlled sprouting on proteins

The number of peptide bonds cleaved by endoprotease activity was indirectly evaluated as a function of the amount of free amino groups by the *o*-phthalaldehyde (OPA) method according to Perri *et al*. (*30*) measuring the absorbance at 340 nm (spectrophotometer model V-730; JASCO) using serine as a calibration standard. The results were expressed as µmol serine per mg protein. The changes in protein extractability under reducing and non-reducing conditions were measured by size exclusion high-performance liquid chromatography (SE-HPLC) using an LC-2010 system (Shimadzu) equipped with a Biosep-SEC-S4000 column (pore size 500 Å; Phenomenex) following the method described by Nivelle *et al.* (*31*). Protein extractability in an SDS-containing medium under non-reducing conditions was calculated from the area under the SE-HPLC chromatogram of a sample. Protein extractability was later expressed as a percentage of the total area obtained when the samples were extracted under reducing conditions. In addition, the SE-HPLC profiles were divided into four fractions according to the protein classification proposed by Ohm *et al*. (*32*): F1 (5.0–7.1 min), F2 (7.1–9.2 min), F3 (9.2–10.6 min) and F4 (10.6–14.0 min). Peak area in % of each eluted protein fraction was calculated based on the total absorbance area.

### Pasting properties of flour suspension

A Rapid Visco Analyzer instrument (RVA series 4500; Perten Instruments, PerkinElmer, Shelton, CT, USA) was used to prepare the flour suspensions and follow the apparent viscosity profile of the samples as a function of temperature and time. To carry out the assay, 5 g of flour (14 % moisture basis) were suspended in 25 mL of deionised water and placed into the aluminium canisters. The RVA 01.05 Method (*33*) was used. The parameters recorded were peak viscosity, peak time, final viscosity, breakdown viscosity and setback viscosity.

### Effect of controlled sprouting on bioactive compounds and phytic acid

As reported by Bustos *et al*. (*34*), total polyphenols were extracted from unsprouted and sprouted whole-wheat flour by mixing 1 g of flour sample with 5 mL of *φ*(acetone,water)=70 % and 0.1 % HCl. Free phenolic acids were determined colorimetrically using the Folin-Ciocalteu method (*35*), and the results were expressed as mg gallic acid equivalents per 100 g. The reducing activity was determined using the Fe(III) ion reducing antioxidant power (FRAP) assay and ABTS˙^+^ scavenging activity according to Podio *et al*. (*36*), and the results were expressed as mg of Trolox equivalents per 100 g. The phosphorus content of phytic acid was determined according to Haug and Lantzsch photometric method modified by Raboy *et al.* (*37*). Dodecasodium phytate was used for calibration and the results were expressed as mg of phosphorus per g of phytic acid.

### Statistical analysis

Statistical analysis was performed using InfoStat software v. 1.0 (*38*). The results were analysed using ANOVA and compared using Di Rienzo, Guzmán and Casanoves (DGC) multiple comparison test at a significance level of 0.05 to determine significant effects of sprouting temperature and time on response variables.

## RESULTS AND DISCUSSION

### Effect of sprouting time and temperature on the degree of sprouting

**Fig. 1:**
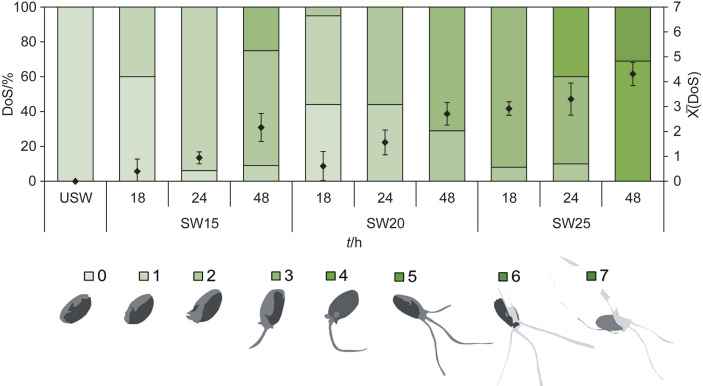
Effect of controlled conditions (temperature of 15, 20 or 25 °C and 18, 14 or 48 h of sprouting) on the degree of sprouting (DoS) of wheat grains and their average degree of sprouting, indicating the visible length of the coleoptile and radicles. The average degree of sprouting is represented with diamonds. USW=unsprouted wheat, SW=sprouted wheat

The fractions of the grains with different degrees of sprouting are denoted with numbers as shown in Fig. 1. After 18 h, differences in sprouting process at various temperatures were visible, but only 50 % of the grains had sprouted at 15 and 20 °C. Sprouting at 25 °C for 18 h resulted in the same average degree of sprouting (DoS=2–3) as that reached at 15 and 20 °C after 48 h, which indicates an already developed embryo emerging from the seed coat. In particular, the sprouting process at 25 °C was the most pronounced because it favoured the increase in the metabolic rate in the grains. After 24 and 48 h of incubation at this temperature, about 40 and 100 % of the grains, respectively, showed a DoS>4, characterised by radicles longer than a whole grain. Although sprouting was faster under these conditions, it was less controlled and led to greater changes in the grain. Moreover, according to the Whole Grains Council (*16*), even these grains could no longer be considered whole grains. The metabolic rate of grains sprouted at 15 and 20 °C decreased considerably, although these conditions led to more homogeneous sprouting.

### Effect of controlled sprouting on starch

During sprouting, α-amylases were synthesised *de novo* in the wheat grains, leading to degradation of starch into dextrins and short-chain carbohydrates with reducing potential (Table 1). The investigated sprouting conditions have a significant effect on α-amylase activity, as found in other studies (*39*, *40*). A very low α-amylase activity was found in unsprouted whole-wheat flour (0.14 AU/g). This activity increased significantly (p<0.05) during controlled sprouting, between 3 and 30 times, and sprouting temperature had the strongest effect. The α-amylase activity followed a linear trend with DoS (r=0.97, p<0.05). Sprouting at 15 °C for 18 h was not sufficient to increase α-amylase activity, but after 24 and 48 h of sprouting, this activity increased 3 and 15 times, respectively, compared to unsprouted whole-wheat flour. In addition, these samples showed a slight decrease in starch mass fraction of about 10 % and an increase in the mass fraction of reducing sugars of 40–60 %. This increasing trend with sprouting time was also observed at 20 °C, which promoted α-amylase activity up to 15 times more than in unsprouted whole-wheat flour. As a result, a 12–15 % reduction of starch mass fraction and a 50–90 % increase in the reducing sugars were observed. The rate of increase in α-amylase activity at 25 °C was practically twice as high as that obtained at 15 °C. The samples of whole-wheat flour sprouted at 15 and at 20 °C for 48 h showed equivalent α-amylase activities. Sprouting at 25 °C for 48 h caused the highest increase in α-amylase activity (3.92 AU/g), which is 30 times higher than that of unsprouted whole-wheat flour. The degradation of starch mass fraction reached up to 30 %, while the release of reducing sugars was up to 3 times higher than in the unsprouted whole-wheat flour.

**Table 1:** Proximal composition and enzymatic activity of USWF and SWF obtained under different sprouting conditions

	Sprouting conditions				*w*/%				α-amylase activity/(AU/g)	Xylanase activity/ (XU/g)	Free amino acid groups in protein as *b*(serine)/ (µmol /mg)
Sample	Temperature/°C	*t*/h	Ash	Protein	Lipid	Starch	Reducing sugars	Water-soluble pentosan
USWF			(1.59±0.03)^a^	(12.32±0.03)^a^	(2.22±0.05)^a^	(67.2±0.4)^e^	(2.04±0.09)^a^	(0.61±0.02)^a^	(0.14±0.01)^a^	(0.28±0.01)^a^	(0.19±0.00)^a^
SWF	15	18	(1.68±0.09)^a^	(12.36±0.05)^a^	(2.2±0.1)^a^	(63.3±2.1)^d^	(2.1±0.2)^a^	(0.81±0.04)^a^	(0.19±0.01)^a^	(0.29±0.02)^a^	(0.28±0.01)^b^
	15	24	(1.82±0.03)^b^	(12.2±0.4)^a^	(2.30±0.06)^b^	(60.8±1.7)^c^	(2.8±0.2)^b^	(1.1±0.1)^b^	(0.40±0.03)^b^	(0.52±0.02)^c^	(0.30±0.00)^c^
	15	48	(1.80±0.03)^b^	(12.5±0.1)^a^	(2.39±0.05)^b^	(60.0±1.8)^c^	(3.2±0.3)^b^	(1.26±0.01)^c^	(2.15±0.01)^e^	(0.76±0.06)^f^	(0.45±0.00)^f^
	20	18	(1.78±0.04)^b^	(12.66±0.08)^b^	(2.14±0.06)^a^	(58.8±1.1)^c^	(3.0±0.2)^b^	(1.12±0.00)^b^	(0.65±0.09)^c^	(0.44±0.03)^b^	(0.31±0.00)^c^
	20	24	(1.78±0.01)^b^	(12.9±0.2)^b^	(2.32±0.05)^b^	(56.0±1.0)^b^	(3.04±0.07)^b^	(1.3±0.1)^c^	(1.26±0.05)^d^	(0.59±0.02)^d^	(0.35±0.01)^d^
	20	48	(1.75±0.07)^b^	(12.78±0.09)^b^	(2.45±0.05)^b^	(57.8±1.5)^c^	(3.9±0.1)^c^	(1.40±0.00)^c^	(2.0±0.1)^e^	(0.84±0.03)^g^	(0.48±0.01)^g^
	25	18	(1.7±0.01)^a^	(12.0±0.1)^b^	(2.15±0.09)^a^	(54.5±1.8)^b^	(3.77±0.04)^c^	(1.11±0.00)^b^	(2.82±0.00)^f^	(0.37±0.03)^b^	(0.41±0.00)^e^
	25	24	(1.78±0.01)^b^	(13.14±0.07)^c^	(2.35±0.05)^b^	(55.3±0.6)^b^	(4.2±0.3)^c^	(1.55±0.08)^d^	(2.92±0.09)^f^	(0.68±0.03)^e^	(0.44±0.01)^f^
	25	48	(1.79±0.00)^b^	(13.22±0.04)^c^	(2.57±0.03)^c^	(48.1±1.2)^a^	(5.38±0.06)^d^	(1.7±0.2)^d^	(3.92±0.03)^g^	(0.82±0.00)^g^	(0.40±0.00)^e^
MS (temperature)			0.001	1.48*	0.01	228.35*	9.19*	0.47*	18.22*	0.10*	0.02*
MS (time)			0.02	0.14	0.27*	39.56*	4.70*	0.57*	7.39*	0.17*	0.04*

Different letters in superscript within a column indicate significant differences according to the Di Rienzo, Guzmán and Casanoves (DGC) test (p≤0.05). *p<0.05. USWF=unsprouted whole-wheat flour, SWF=sprouted whole-wheat flour, MS=mean of squares, XU=xylanase unit, AU=amylase unit

The sugar composition of flour samples was affected by sprouting conditions (Table 2). In general, glucose and sucrose mass fractions were significantly higher in all sprouted whole-wheat flour samples than in the unsprouted whole-wheat flour and followed similar patterns. Sucrose mass fraction was positively associated with higher DoS and α-amylase activity. Regardless of temperature, longer sprouting times (48 h) favoured the increase in maltose mass fraction, while fructose mass fraction increased 4 times only when sprouting occurred at 25 °C for 48 h. Relative abundance of each sugar remained constant among samples. Sucrose was the predominant sugar and accounted for about 60 % of total sugar content. Benincasa *et al*. (*8*) also reported different effects depending on sprouting time, with sucrose as the major carbohydrate source in the early stage of wheat sprouting (24–48 h). This is because sucrose plays a crucial role in carbohydrate transport during early wheat sprouting (*40*). Different sprouting conditions resulted in different sugar composition in sprouted whole-wheat flour samples, which could affect yeast fermentation in the dough and reduce fermentation time. Additionally, a more diverse sugar composition could add natural sweetness to products when sprouted wheat flour is used, which could help manufacturers reduce the amount of added sugar in wheat-based products. In whole-wheat bread, bitterness usually remains at the end of mastication and can be considered a negative attribute (*12*). Therefore, a higher content of reducing sugars in sprouted whole-wheat flour samples may limit or mask the perception of bitterness.

**Table 2:** Sugar composition determined by HPLC-IR

	Sprouting condition		*w*(sugar composition)/%			
Sample	Temperature/°C	*t*/h	Fructose	Glucose	Sucrose	Maltose
USWF			(0.03±0.00)^a^	(0.01±0.00)^a^	(0.46±0.00)^a^	(0.19±0.00)^a^
SWF	15	18	(0.07±0.02)^a^	(0.17±0.04)^b^	(0.73±0.05)^b^	(0.16±0.03)^a^
	15	24	(0.05±0.01)^a^	(0.17±0.09)^b^	(0.70±0.02)^b^	(0.13±0.00)^a^
	15	48	(0.05±0.01)^a^	(0.29±0.03)^b^	(0.99±0.03)^c^	(0.30±0.01)^b^
	20	18	(0.05±0.00)^a^	(0.17±0.05)^b^	(0.67±0.07)^b^	(0.18±0.04)^a^
	20	24	(0.06±0.02)^a^	(0.21±0.06)^b^	(0.75±0.03)^b^	(0.21±0.05)^a^
	20	48	(0.12±0.05)^a^	(0.18±0.03)^b^	(1.33±0.04)^d^	(0.34±0.03)^b^
	25	18	(0.09±0.01)^a^	(0.15±0.06)^b^	(0.96±0.02)^c^	(0.23±0.01)^a^
	25	24	(0.10±0.01)^a^	(0.24±0.00)^b^	(1.33±0.01)^d^	(0.32±0.01)^b^
	25	48	(0.17±0.10)^b^	(0.4±0.2)^c^	(1.6±0.2)^e^	(0.41±0.07)^c^
MS (temperature)			0.01	0.02	0.77*	0.04*
MS (time)			0.01	0.05	0.83*	0.09*

Different letters in superscript within a column indicate significant differences according to the DGC test (p≤0.05). *p<0.05. USWF=unsprouted whole-wheat flour, SWF=sprouted whole-wheat flour, MS=mean of squares

**Fig. 2:**
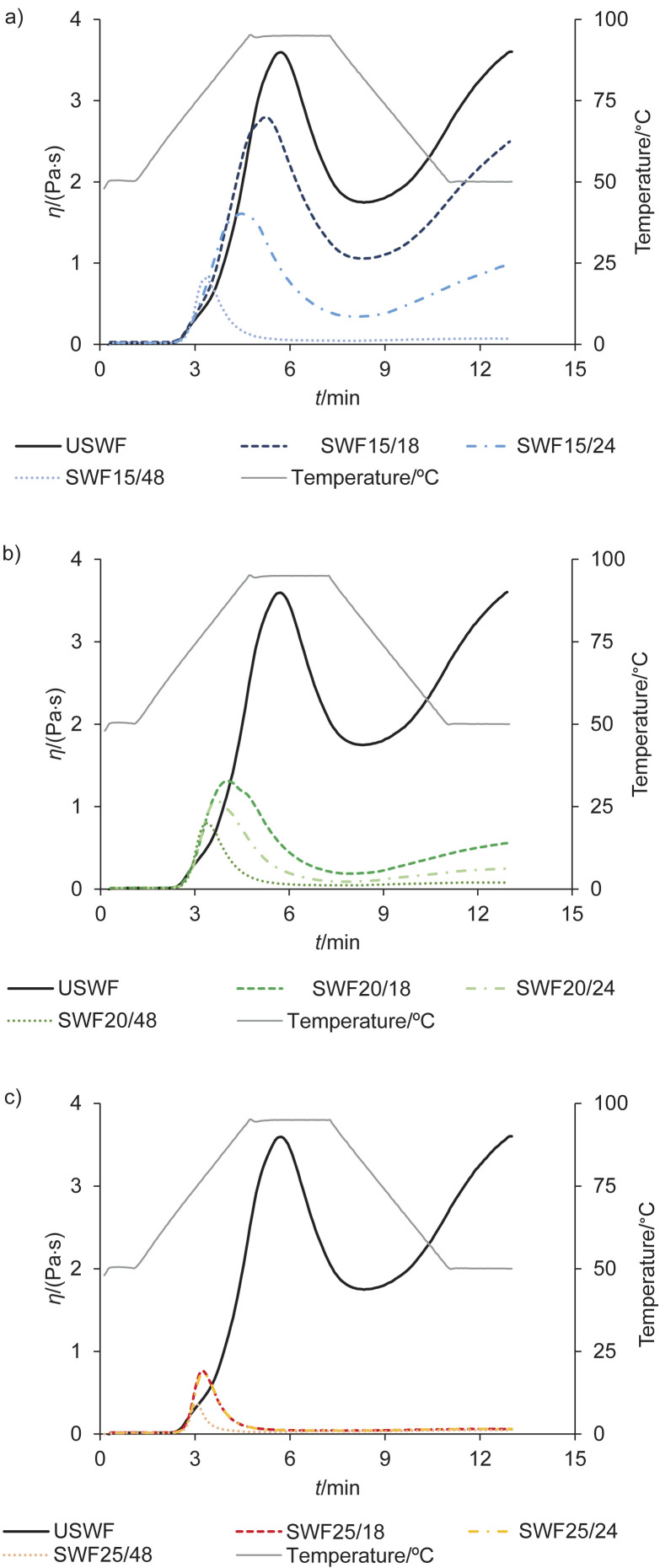
Pasting properties of unsprouted whole-wheat flour (USWF) and sprouted whole-wheat flour (SWF) suspensions obtained at different temperatures: a) 15, b) 20 and c) 25 °C for 18, 24 and 48 h

The starch hydrolysis resulting from the α-amylase activity also had an effect on the pasting properties of the flour suspensions (Fig. 2). Simultaneously with the increase in α-amylase activity, a significant reduction in viscosity was observed during both heating (peak viscosity) and cooling (final viscosity) steps, which led to a considerable decrease in the swelling, gelatinisation and gelation properties of sprouted whole-wheat flour. Already after 18 h of sprouting at 15 °C, the peak viscosity of the suspension of sprouted whole-wheat flour was found to decrease by almost 25 %, while it decreased by up to 80–90 % in suspensions of sprouted whole-wheat flour obtained after 48 h of sprouting. A temperature of 25 °C had the greatest influence on the structure of the starch during sprouting. Grassi *et al.* (*41*) found similar trends in the decrease of viscosity, which could be mainly due to the lower mass fraction of starch, as α-amylases break the α-1,4 glycosidic bonds between the glucose molecules in the chains during sprouting and then degrade the starch granules during the pasting step (*42*). The peak time for all sprouted whole-wheat flour suspensions was observed to decrease by about 50 % with increasing α-amylase activity. The breakdown viscosity, final viscosity and setback viscosity values of all sprouted whole-wheat flour suspensions showed similar changes with the progression of sprouting. In general, the changes in the characteristic shape of the RVA curves of sprouted whole-wheat flour suspensions became more pronounced with increasing DoS of the grains and more linear in the final stage of the test. According to Silva Oliveira *et al.* (*42*), the increase in hydrolysed amylose and amylopectin molecules led to fewer interactions between the starch chains. Therefore, high temperatures and long incubation times caused changes in the starch structure that could negatively affect the functionality of the flour. Conversely, sprouted whole-wheat flour obtained under milder sprouting conditions with moderate α-amylase activity could favour the development of loaf volume before the crumb structure settles during breadmaking when these flour types are added to the bread formulation. Moreover, the production of maltodextrins through the action of α-amylase could have an antistaling effect on wheat bread crumb by reducing amylopectin retrogradation when enriched with sprouted whole-wheat flour (*12*). Consequently, the enrichment with the sprouted whole-wheat flour instead of conventional improvers, *i.e.* enzymatic improver, malt or maltogenic α-amylase, could improve breadmaking performance of certain flour types.

### Effect of controlled sprouting on non-starchy polysaccharides

Besides starch, non-starch polysaccharides of bran also play an important structural and functional role in cereal grains. The results in Table 1 show that the selected sprouting conditions significantly (p<0.05) favoured the increase in endoxylanase activity and followed a linear trend with DoS. Cell wall degradation is a critical step in sprouting, as these cell walls form a physical barrier for α-amylases and proteases to reach and degrade their intracellular starch and protein substrates. However, endoxylanase activity increases slowly in the first 48 h compared to the increase in α-amylase activity because these enzymes are often produced late in the sprouting process (*41*). The endoxylanase activity of unsprouted whole-wheat flour was low (0.29 XU/g). As reported by Benincasa *et al*. (*8*), controlled sprouting conditions increased endoxylanase activity between 1.3 and 3 times compared to unsprouted whole-wheat flour, with the exception of the sprouting at 15 °C during 18 h (Table 1). Consequently, insoluble pentosans were hydrolysed, which led to a two- to threefold increase in water-soluble pentosans (Table 1). This finding is of great importance for whole-wheat products because it does not interfere with the gluten network. The degradation of the xylan backbone affects the molecular mass, solubility and physicochemical properties of pentosans (*11*). Cardone *et al.* (*13*) obtained comparable results in sprouted cereals. Thus, sprouted whole-wheat flour with sufficient xylanase activity can be used as a substitute for commercial microbial xylanases, which are often added to improve the processing parameters and the quality of wheat-based products such as crumb elasticity and softness. In addition, this sprouted whole-wheat flour could also improve the nutritional profile of the products as the non-starch polysaccharides belong to the dietary fibre fraction (*43*).

### Effect of controlled sprouting on proteins

A slight increase (5–10 %) in protein mass fraction was observed after sprouting at 20 and 25 °C compared to unsprouted whole-wheat flour. Similarly, sprouted whole-wheat flour samples showed a slight increase in ash (10–15 %) and lipid mass fraction (5–15 %) after 24 h of sprouting compared to unsprouted whole-wheat flour, regardless of temperature. However, as observed by Olaerts *et al.* (*44*), these increases were due to the loss of matter through respiration and starch hydrolysis (Table S1).

In addition to the changes in starch and non-starch molecules due to sprouting, the protein matrix was also affected by endoproteolytic enzymes. Proteases break down high-molecular-mass proteins into smaller subfractions and free amino acid groups, which are crucial for cell metabolism (*45*). However, the degradation of proteins in wheat flour plays an important role in the deterioration of breadmaking quality, even more than starch (*12*), because sprouting impairs flour functionality by reducing dough elasticity and strength (*14*). During sprouting, the amount of free amino groups increased 2–3 times compared to unsprouted whole-wheat flour. A high degree of proteolysis was observed after prolonged sprouting (48 h) at 20 °C. These results are in agreement with previous studies by Koehler *et al.* (*46*) and Žilić *et al.* (*39*). Koehler *et al.* (*46*) reported that gliadins were strongly degraded at 20 °C, while glutenins were affected at 25 °C. Moreover, Naumenko *et al*. (*47*) showed that significant gluten decomposition required sprouting times longer than 100 h.

**Fig. 3:**
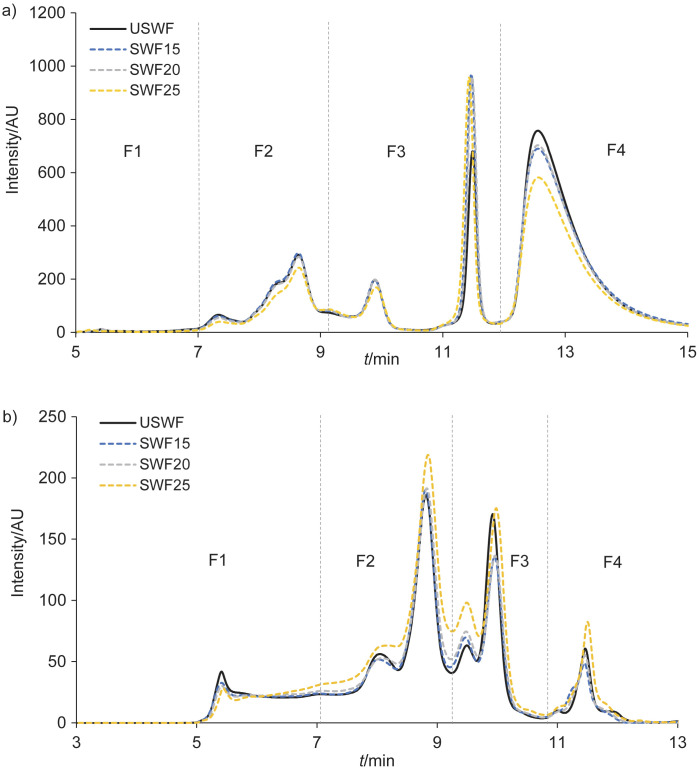
Size-exclusion HPLC chromatograms of flour proteins extracted from unsprouted whole-wheat flour (USWF; full black lines) and sprouted whole-wheat flour (SWF; dotted coloured lines) obtained after 48 h at 15, 20 and 25 °C under: a) reducing and b) non-reducing conditions. AU=arbitrary unit

**Fig. S1:**
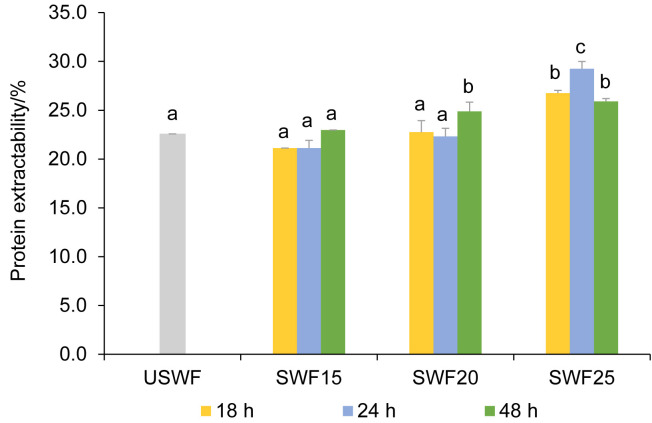
Protein extractability in Sodium Dodecyl Sulfate (SDS) containing medium under non-reducing conditions at 15, 20 and 25 °C compared to unsprouted whole-wheat flour (USWF)

SE-HPLC chromatograms showed qualitative changes in the molecular mass distribution of the proteins extracted in the buffer containing SDS under reducing and non-reducing conditions (Fig. 3a and Fig. 3b). A slight increase (15–30 %) in protein extractability compared to unsprouted whole-wheat flour extracts was observed (Fig. S1) when sprouting was performed at 20 °C for 48 h and 25 °C regardless of time. This could indicate incipient changes in protein structure due to proteolytic activity. Additionally, under both reducing and non-reducing conditions (after all bonds were broken), a small shift from high-molecular-mass proteins to low-molecular-mass proteins was observed during sprouting (Fig. 3a and Fig. 3b). Simsek *et al*. (*48*) observed similar results after wheat sprouting for 48–72 h. After 48 h of sprouting at 20 °C and 18 h at 25 °C, the peak area of high-molecular-mass proteins, the main components of F1, was reduced, by about 30 %. However, when converted to percentage of total peak area (Table S2), the F1 fraction of the extracted proteins under reducing conditions represented only a small percentage of the total area; this was probably due to the limited discriminatory power of the analysis with respect to the fractions that eluted first (*44*). As observed by Ohm *et al*. (*32*), the reduction in F1 area may indicate the degradation of glutenin. This is critical as high-molecular-mass proteins are essential for gluten strength, especially in whole-wheat products where the gluten matrix is diluted by the presence of bran and germ (*49*). However, Cardone *et al*. (*13*) reported that the decrease in gluten aggregation properties in sprouted whole grain flour did not have negative effect on breadmaking performance. This was attributed to the unique protein aggregations found in their study. The gliadin (F2) peak area percentages of sprouted whole-wheat flour extracts did not change, indicating that it did not degrade. As a result of the degradation of the F1 fraction, an peak area percentage (F3), which corresponds to lower-molecular-mass protein fragment or albumins, increased slightly during sprouting. In contrast, the peak area percentage corresponding to globulins (F4) did not vary among sprouted whole-wheat flour extracts. Sprouting conditions at 15 °C for 24 h and 20 °C for 18–24 h favoured a moderate increase in α-amylase and xylanase. This preserved essential polymeric proteins of the gluten network, suggesting good breadmaking potential. Additionally, this controlled depolymerisation can favour dough extensibility from the flour with a high ratio of tenacity to extensibility, such as those usually obtained from Argentine wheat, thus reducing mixing time and improving loaf volume (*50*). In addition, it could favour the development of the desirable products of the Maillard reaction (*1*) and improve the protein digestibility (*6*).

### Effect of controlled sprouting on free polyphenol content and antioxidant capacity

Results showed that free phenolic mass fraction increased significantly (20–50 %) after controlled sprouting compared to unsprouted whole-wheat flour, except at 15 °C during 18 h sprouting (Table 3). Sprouting at 20 and 25 °C after only 18 h was sufficient to increase of free polyphenol mass fraction in sprouted whole-wheat flour extracts. In general, free polyphenol mass fraction was more affected by sprouting time than by temperature. The antioxidant activity of sprouted whole-wheat flour extracts was also positively affected by sprouting. Pitzschke *et al*. (*49*) suggested that antioxidants may play a crucial role in breaking seed dormancy and sprouting to scavenge the reactive oxygen species and serve as protection against environmental changes during growth. Sprouting conditions affected the antioxidant capacity values determined by both FRAP and ABTS methods, with more pronounced differences in ABTS˙^+^ values. The highest FRAP values were obtained with 48-hour sprouting at 25 °C, which was approx. 45 % higher than the value observed in unsprouted whole-wheat flour extract. The highest antioxidant capacity values using the ABTS method were also observed in sprouted whole-wheat flour extracts obtained at 20 and 25 °C after 48 h of sprouting, which was approx. 4 times higher than in the unsprouted whole-wheat flour extract. The increase in free polyphenol mass fraction and antioxidant capacity could be related to the release of phenols bound to lignin or arabinoxylans from the cell walls by the activation of endoxylanases. It could also be due to the *de novo* biosynthesis of polyphenols (to maintain homeostasis) and to the increase in the content of free -SH groups in wheat grains during sprouting (*51*). Sprouted whole-wheat flour, which is characterised by moderate enzyme activity, therefore also has a higher nutritional value.

**Table 3:** Free phenolic content, antioxidant capacity and phytic acid content of USWF and SWF

	Sprouting condition					
Sample	Temperature/°C	*t*/h	FPC as *w*(GAE)/ (mg/100 g)	FRAP as *w*(TE)/ (mg/100 g)	ABTS as *w*(TE)/ (mg/100 g)	PA as *w*(PAP)/ (mg/g)
USWF			(64.7±4.3)^a^		(1.2±0.2)^a^	(17.3±0.9)^c^
SWF	15	18	(69.95±0.01)^a^	(1.15±0.03)^a^	(1.46±0.08)^a^	(16.2±0.8)^c^
	15	24	(89.1±2.2)^c^	(1.16±0.03)^a^	(2.6±0.2)^b^	(16.0±1.4)^c^
	15	48	(94.1±2.4)^c^	(1.37±0.04)^b^	(3.50±0.04)^b^	(15.7±0.3)^c^
	20	18	(75.8±5.8)^b^	(1.3±0.1)^b^	(1.37±0.07)^a^	(14.5±0.6)^b^
	20	24	(86.02±8.04)^c^	(1.3±0.1)^b^	(3.0±0.2)^b^	(13.7±0.9)^b^
	20	48	(91.2±2.6)^c^	(1.47±0.03)^b^	(4.5±0.5)^c^	(14.37±0.07)^b^
	25	18	(80.6±1.7)^c^	(1.35±0.01)^b^	(3.10±0.07)^b^	(12.2±0.6)^a^
	25	24	(96.7±4.3)^c^	(1.46±0.04)^b^	(3.0±0.1)^b^	(11.2±0.6)^a^
	25	48	(90.4±1.8)^c^	(1.6±0.1)^c^	(4.6±0.4)^c^	(10.5±0.1)^a^
MS (temperature)			31.51	1791.12*	0.70*	65.25*
MS (time)			278.00*	1652.43*	2.63*	2.11

Different letters in superscript within a column indicate significant differences according to the DGC test (p≤0.05). *p<0.05. USWF=unsprouted whole-wheat flour, SWF=sprouted whole-wheat flour, MS=mean of squares, FPC=free polyphenol content, GAE=gallic acid equivalents, FRAP=Fe(III) reducing antioxidant power, ABTS˙^+^=2,2’-azino-bis(3-ethylbenzothiazoline-6-sulfonic acid), TE=Trolox equivalents, PA=phytic acid, PAP=phosphorus in phytic acid

### Effect of controlled sprouting on phytate content

Another nutritional benefit enhanced by controlled sprouting is the activation of phytases, which are responsible for the release of stored inorganic phosphorus, myoinositol and chelated divalent cations for seedling growth (*10*). The sprouting temperature was a decisive factor for the reduction of phytic acid (Table 3). Sprouting at 20 and 25 °C reduced this antinutrient by 15–20 and 30–40 %, respectively. The results were consistent with those found by Olaerts and Courtin (*9*) in sprouted wheat. In this way, controlled sprouting could lead to a better availability of minerals in wholegrain baked products.

## CONCLUSIONS

The sprouting conditions used in this study affected the physicochemical composition and nutritional profile of the obtained sprouted whole-wheat flour as a result of hydrolytic enzymes activation. In general, a sprouting temperature of 25 °C led to an excessive increase in enzymatic activity and metabolic processes. These conditions strongly affected the associated starch matrix and pasting properties, and to a lesser extent the structure of the cell walls and the protein matrix. Sprouting at 15 and 20 °C for 24 h allowed better control of the process, since it promoted an improvement in nutritional value and provided techno-functional benefits due to the moderate activation of α-amylase and xylanase, making it a better food than the unsprouted wheat grains. Additionally, the gluten proteins were generally not modified. This is crucial for good quality whole-wheat baked products as the gluten is diluted in the presence of bran and germ. In conclusion, sprouted whole-wheat flour, obtained under milder sprouting conditions and with moderate enzymatic activity, can be a potential techno-functional ingredient for formulating wholegrain baked goods with an improved nutritional profile. Its use could partially or fully replace the use of conventional flour improvers, allowing the product to be included in a clean label.

## Appendix

**Table S1:** Effect of controlled sprouting on dry matter losses of initial mass of wheat grains in sprouted whole-wheat flour

Sprouting conditions for sprouted whole-wheat flour		
Temperature/°C	*t*/h	*w*(dry matter loss)/%
15	18	(7.8±2.0)^a^
15	24	(7.8±1.1)^a^
15	18	(8.4±1.5)^a^
20	18	(7.7±2.7)^a^
20	24	(12.2±0.8)^a^
20	48	(9.9±1.6)^a^
25	18	(10.5±2.6)^a^
25	24	(11.4±0.3)^a^
25	48	(14.6±2.9)^b^

Different letters in superscript indicate significant differences according to the DGC test (p≤0.05)

**Table S2:** Size-exclusion HPLC peak area values for proteins extracted from USWF and SWF under reducing and non-reducing conditions

	Sprouting conditions		Non-reducing conditions					Reducing conditions			
Sample			F1	F2	F3	F4		F1	F2	F3	F4
	Temperature/°	*t*/h	Peak area/%								
USWF			(18.6±0.2)^b^	(45.1±1.1)^a^	(27.8±0.6)^a^	(8.5±0.8)^a^		(3.19±0.04)^b^	(23.2±0.7)^a^	(11.2±0.2)^a^	(62.4±1.0)^a^
SWF	15	18	(19.8±1.9)^b^	(45.4±0.3)^a^	(26.5±2.3)^a^	(8.2±0.2)^a^		(2.85±0.01)^b^	(23.3±0.0)^a^	(15.8±0.5)^b^	(58.0±0.5)^a^
	15	24	(18.5±0.5)^b^	(47.2±1.9)^a^	(27.62±0.04)^a^	(6.7±1.5)^a^		(2.780.1)^b^	(23.2±0.7)^a^	(17.1±2.7)^b^	(56.9±1.9)^a^
	15	48	(16.8±1.2)^b^	(44.3±1.4)^a^	(28.6±1.1)^a^	(10.3±1.5)^b^		(2.60.2)^b^	(22.5±1.4)^a^	(15.54±0.07)^b^	(59.4±1.5)^a^
	20	18	(17.4± 0.8)^b^	(46.6±0.7)^a^	(28.3±0.4)^a^	(7.7±0.2)^a^		(2.8±0.2)^b^	(23.2±0.7)^a^	(15.510.8)^b^	(58.5±0.1)^a^
	20	24	(17.2±1.5)^b^	(45.8±0.6)^a^	(29.0±1.8)^a^	(8.0±0.3)^a^		(2.71±0.01)^b^	(23.4±0.1)^a^	(15.8±0.6)^b^	(58.2±0.5)^a^
	20	48	(14.2±0.7)^a^	(47.6±3.3)^a^	(32.0±0.5)^b^	(8.71±0.08)^a^		(1.9±0.2)^a^	(19.2±5.9)^a^	(17.8±0.6)^b^	(61.1±5.9)^a^
	25	18	(15.1±1.7)^a^	(43.4±2.3)^a^	(33.6±3.7)^b^	(8.0±0.4)^a^		(2.2±0.2)^a^	(23.39±0.07)^a^	(18.2±0.7)^b^	(55.6±1.0)^a^
	25	24	(15.64±0.06)^a^	(45.93±0.05)^a^	(29.41±0.02)^a^	(9.0±0.1)^a^		(2.1±0.2)^a^	(23.3±0.5)^a^	(19.8±1.5)^b^	(55.4±0.9)^a^
	25	48	(20.74±0.05)^b^	(43.4±0.3)^a^	(27.11±0.07)^a^	(8.7±0.2)^a^		(2.2±0.1)^a^	(25.5±1.1)^a^	(12.4±0.7)^a^	(59.9±0.3)^a^

F1 to F4 are peak areas presented in Fig. 3. Different letters in superscript within a column indicate significant differences according to the DGC test (p≤0.05). USWF=unsprouted whole-wheat flour, SWF=sprouted whole-wheat flour.
